# Thermoluminescent and Monte Carlo dosimetry of IR06‐P103d brachytherapy source

**DOI:** 10.1120/jacmp.v12i4.3581

**Published:** 2011-11-15

**Authors:** Pooneh Saidi, Mahdi Sadeghi, S. Hamed Hosseini, Claudio Tenreiro

**Affiliations:** ^1^ Department of Nuclear Engineering, Science and Research Branch Islamic Azad University Tehran Iran; ^2^ Agricultural, Medical and Industrial Research School Nuclear Science and Technology Research Institute Karaj Iran; ^3^ Department of Energy Science Sungkyunkwan University 300 Cheongcheon‐dong Suwon Korea

**Keywords:** P103d, thermoluminescent dosimetry, Monte Carlo, brachytherapy

## Abstract

This work presents experimental dosimetry results for a new P103d brachytherapy seed, in accordance with the AAPM TG‐43U1 recommendation that all new low‐energy interstitial brachytherapy seeds should undergo one Monte Carlo (MC) and at least one experimental dosimetry characterization. Measurements were performed using TLD‐GR200A circular chip dosimeters using standard methods employing thermoluminescent dosimeters in a Perspex phantom. The Monte Carlo N‐particle (MCNP) code, version 5 was used to evaluate the dose‐rate distributions around this model P103d source in water and Perspex phantoms. The consensus value for dose‐rate constant of the IR06‐P103d source was found equal to $0.690\;\text{cG}\cdot h^{-1}\cdot U^{-1}$. The anisotropy function, F(r, θ), and the radial dose function, $g_{L}(r)$, of the seed were measured in Perspex phantom and calculated in both Perspex and liquid water phantom. The measured values were also found in good agreement with corresponding MC calculations.

PACS number: 87.53.Jw

## I. INTRODUCTION

The isotopes ${\;}^{125}I$ and P103d are frequently used in low‐dose rate (LDR) brachytherapy sources. Common tumor sites treated with such materials are prostate and eye. Prostate brachytherapy with ${\;}^{125}I$ and P103d sources is a treatment of choice for patients with early‐stage prostate cancer. P103d is used because of its low energy emissions (20 keV), which allow for a rapid decrease in dose with distance, and also because of the short half‐ life (17 days) results in higher dose rates than ${\;}^{125}I$.^(^ ^1^ ^–^ ^3^ ^)^ With the increasing demand for P103d seed sources for permanent prostate implantation, new sources have been introduced and applied.^(^ ^2^ ^)^ According to American Association of Physicists in Medicine (AAPM) Radiation Therapy Committee recommendation, the dosimetry characteristics for all new interstitial brachytherapy seeds of energies less than 50 keV should be established by two independent investigators, theoretical calculations, and experimental measurements.^(^ ^4^ ^,^ ^5^ ^)^ Accordingly this work reports thermoluminescent dosimeter (TLD) measurements for the dosimetric characteristics of the IR06‐P103d brachytherapy seed. We used TLD‐GR200A thermoluminescent dosimeters (TLDs) and two Perspex phantoms, one for the anisotropy function, F(r, θ), and the other for the radial dose function, $g_{L}(r)$ measurements. Our work also used Monte Carlo simulations to evaluate between calculated and measured values. Finally, the IR06‐P103d dosimetric data have been compared with data for other P103d commercial sources.

## II. MATERIALS AND METHODS

### A. P103d source description

**Figure 1:**
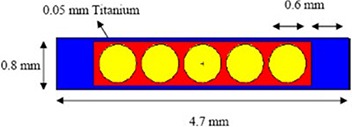
Schematic diagram of the IR06‐P103d seed.

The production of P103d is carried out via the ${\;}^{103}\text{Rh}$(p,n)P103d reaction. After cyclotron production and chemical separation process of P103d, produced palladium‐103 is then absorbed in Amberlite IR‐93 resin (20–50 mesh) beads to encapsulate inside the titanium capsule. Figure 1 shows a schematic diagram of the IR06‐P103d seed. The seed contains five resin beads, each of diameters 0.6 mm with the compositions (by weight percent): H, 8%; C, 90%; N, 0.3%; Cl, 0.7%; and Pd, 1%; and density $1.14\;g/{\text{cm}}^{3}$. The beads are packed inside a titanium cylinder 4.7 mm in length having 0.7 and 0.8 mm internal and external diameter, respectively, and 0.6 mm thick end caps. The active source length is 3 mm.

### B. Thermoluminescent dosimeters (TLD)

TLD‐GR200A (LiF: Mg, Cu, P) (PTW, Freiburg, Germany) circular chips^(^ ^6^ ^)^ of dimension 0.8 mm thickness by 4.5 mm diameter were used in this study. The irradiated TLDs were read using a KFKI RMKI TLD reader (KFKI Research Institute of the Hungarian Academy of Sciences, Budapest, Hungary) and were annealed by heating at 240°C for 10 min followed by fast cooling.

TLD measurement methodology followed the following equation:

(1) $$\frac{\dot{D}(r,\theta )}{S_{K}}=\frac{R_{\det}(r,\theta )\cdot g(t)}{S_{K}\cdot \varepsilon_{\lambda }\cdot E(r,\theta )}$$

where $R_{\det}(r,\theta )$ is TLD reading, *g(T)* is decay correction and is equal to 1/effective exposure time, ${ɛ}_{\lambda }$ is measured response for calibrated beam, and E(r,θ) is TLD response.^(^ ^7^ ^)^

TLD responses have been corrected for background by subtracting the average response of background TLDs from the responses of all other TLDs in each measurement. The sensitivity correction due to physical differences between the TLD chips for each TLD has been obtained by simultaneously irradiating the TLDs. So before every experiment, the entire batch of TLDs was exposed to a calibrated ${\;}^{60}\text{Co}$ standard beam, with a constant dose given each time. The variation of response of the TLDs to the same exposure was tracked by normalizing the individual TLD readings to the average value of TLD readings.

### C. Phantoms

To obtain the radial dose function, g(r), and the anisotropy function, F(r, θ), 30cm×30cm×15cm phantoms of Perspex slabs with composition (by weight percent): H, 8%; C, 60%; O, 32%, and density equal to $1.19\;g/{\text{cm}}^{3}$ were used. The two phantom designs for the measurement of radial dose and anisotropy functions were based on those of Meigooni et al. and Hosseini et al.^(^ ^8^ ^,^ ^9^ ^)^

**Figure 2:**
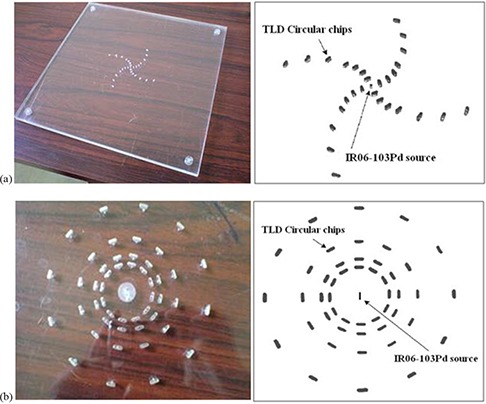
Central slabs of Perspex phantoms used for the experimental determination of: (a) radial dose function values, (b) anisotropy function values.

Figure 2(a) presents the first phantom which is used for the experimental determination of the IR06‐P103d radial dose function values. Holes were drilled in the central phantom slab to accommodate the TLD circular chips so that their circular surface is perpendicular to the slab plane and thus parallel to the source long axis. The measurements were performed at distances of r=0.5,1,1.5,2,3,4 and 5 cm relative to the seed center in the spiral configuration, to minimize the interference of any one TLD with regard to the response by other TLD chips.^(^ ^10^ ^,^ ^11^ ^)^ A total of 28 TLDs (four at each radial distance) can be used in a single experiment because, due to the configuration of the phantom, measurements do not suffer by shadowing effects. The experiment was repeated several times (at least twice) to improve the statistical quality of the data.

The second phantom (Fig. 2(b)) was used for the measurement of the anisotropy function of the IR06‐P103d seed. It has the same geometry and dimensions as the first phantom but differs in the configuration of the source in that it is placed with its long axis parallel to the central slab plane.

The TLDs lie at radial distances of r=1.5,2,3 and 5 cm relative to the seed center, and polar angles θ ranging from 0° to 330° in 30° increments with respect to the seed long axis. The measurements were performed with 48 holes containing TLDs, since it was found that, for the experimental anisotropy function determination at a specific point, shadowing effects due to the TLDs that lie at the same polar angle do not affect results. This is due to the definition of anisotropy function that normalizes dose rate at a particular (r, θ) point to the dose rate at the corresponding point along the transverse source bisector (r, 90°). Therefore, since shadowing was found similar at any polar angle for the same radial distance, the overall effect is cancelled out in the calculation of an anisotropy function.^(^ ^12^ ^)^

### D. Monte Carlo evaluation

Brachytherapy dose distributions were simulated with the version 5 of the Monte Carlo (MC) Radiation Transport Code written by Los Alamos National Laboratory.^(^ ^13^ ^)^ The MCPLIB04 photon cross‐sectional library was applied using data from ENDF/B‐VI.^(^ ^14^ ^)^ The results from MCNP5 calculations can be output using numerous flexible tallies: surface current and flux, volume flux (track length), point or ring detectors, particle heating, fission heating, pulse height tally for energy or charge deposition, mesh tallies, and radiography tallies. Particle fluence and cell‐heating tallies, F4 and F6 respectively, were employed to calculate kerma and absorbed dose in this study. The P103d photon spectrum used in these simulations was obtained from TG‐43U1 Table XIII.^(^ ^4^ ^)^ For the calculations, titanium characteristic X‐ray production was suppressed with δ=5keV (δ is the energy cutoff).^(^ ^15^ ^)^ The spherical Perspex phantom was modeled with a 30 cm diameter, large enough to consider effects of the surrounding medium. The composition of the Perspex taken to be H, 8%; C, 60%; O, 32%, with a density of $1.19\;g/{\text{cm}}^{3}$.^(^ ^2^ ^)^ The seed was positioned at the center of the phantom and an array of detector rings were defined in this phantom for Monte Carlo simulation at polar angles from 0° to 90° in 10° increments and at radial distances of r=0.25,0.5,1,1.5,2,3,4, and 5 cm. According to TG‐43 recommendation, the medium for clinical dose calculations is water, so the dose distribution of the seed was calculated in Perspex and water. The atomic ratio for water is of 2:1 for H:O and $\rho =0.998\;g/{\text{cm}}^{3}$. A simulation was performed for absorbed doses to water in a Perspex medium in order to obtain data comparable with the TLD measurements.

According to TG43‐U1 recommendation, the proposed formula for two‐dimensional dose rate is:

(2) $$\dot{D}(r,\theta )=S_{K}\Lambda \frac{G(r,\theta )}{G(r_{0},\theta_{0})}g(r)F(r,\theta )$$

where $\dot{D}(r,\theta )$ is the dose rate in water at the distance r in cm from a line source and θ denotes the polar angle specifying the point of interest, $S_{k}$ is the air kerma strength has in unit of $U=cGy\;cm^{2}\;h^{-1},\;\Lambda$ is the dose rate constant expressed in $cGy\;h^{-1}\;U^{-1};\frac{G(r,\theta )}{G(r_{0},\theta )}$ is the geometry factor; $r_{0},\theta_{0}$ are the reference positions $r_{0}=1\;\text{cm}$ and $\theta_{0}=90{}^{\circ};g(r)$ is the radial dose function; and F(r,θ) is the anisotropy function.

The dose rate constant, Λ, for the IR06‐P103d seed was calculated as the ratio of the dose to water at 1 cm from the seed along the transverse axis, to the source's air kerma strength $S_{K}$. The air kerma strength was calculated using the recommended equation below:^(^ ^4^ ^)^

(3) $$S_{K}={\dot{K}}_{\delta }(r)r^{2}$$

Due to the low energy of the photons from P103d, it was assumed in the Monte Carlo calculations that all electrons generated by the photon collisions are absorbed locally, so it was assumed that dose is equal to kerma at all points of interest.^(^ ^1^ ^,^ ^2^ ^)^ The air kerma rate, ${\dot{K}}_{\delta }(r)$, of the IR06‐P103d seed was estimated by calculating the dose in 1 mm thick air‐filled rings in vacuum. The rings were bounded by 86° and 94° conics, and defined with a radial increment of 5 cm to 150 cm along the transverse axis of the source to find the $S_{K}$ that is independent to distance.^(^ ^16^ ^)^ The dose distributions were calculated from the energy deposition averaged over a cell tally F6 in MeV/g/source photon.

The MCNP simulation method in this work was benchmarked with Theragenics model 200 source (TheraSeed) (Theragenics Corp., Buford, GA).

The geometry function takes into account the effect of the distribution of radioactive material inside the capsule on the dose distribution.^(^ ^15^ ^)^ G(r,θ) is a function of r which is equal to distance from the source center, polar angle 9, and L, the effective length of source.^(^ ^17^ ^,^ ^18^ ^)^ Geometry function is calculated according to the following equation:

$$G_{p}(r,\theta )=r^{-2}\;(\text{for}\;\text{the}\;\text{point‐source})$$

$$G_{L}(r,\theta )=\left\{\begin{array}{c} \frac{\beta }{Lr\sin\theta }\\ {\left(r^{2}-\frac{L^{2}}{4}\right)}^{-1} \end{array}\right\}\begin{array}{l} if\;\theta \neq 0^{0}\\ if\;\theta =0^{0} \end{array}\;(\text{for}\;\text{the}\;\text{line‐source})$$

(4) $$\beta =\tan^{-1}(\frac{r\sin\theta }{r\cos\theta -L/2})-(\frac{r\sin\theta }{r\cos\theta +L/2})$$

The radial dose function describes the effect of tissue attenuation on photons emitted from a brachytherapy source and accounts for the dose falloff along the source transverse axis due to the photon scattering and attenuation. According to the TG‐43U1 Monte Carlo methodology, g(r) was calculated by using line‐source (with an effective length of 3 mm) and point‐source geometry for IR06‐P103d seed.^(^ ^4^ ^)^

(5) $$g_{X}(r)=\frac{\dot{D}(r,\theta_{0})}{\dot{D}(r_{0},\theta_{0})}\frac{G_{L}(r_{0},\theta_{0})}{G_{L}(r,\theta_{0})}$$

Dose variations due to the distribution of radioactivity within the seed, self‐absorption and oblique filtration of the radiation in the encapsulating material are described by the 2D anisotropy function.

(6) $$F(r,\theta )=\frac{\dot{D}(r,\theta )}{\dot{D}(r,\theta_{0})}\frac{G_{L}(r,\theta_{0})}{G_{L}(r,\theta )}$$

As TG‐43U1 recommends a single consensus dataset for each source model, the consensus values for dose rate constant of the IR06‐P103d seed has been obtained in this study. The consensus data were defined as the ideal candidate dataset having the highest resolution, covering the largest distance range, and having the highest degree of smoothness. The consensus dose rate constant value should be obtained by the averaged experimental and Monte Carlo Λ values:

(7) $${\;}_{CON}\Lambda =\frac{{\;}_{EXP}\Lambda +{\;}_{MC}\Lambda }{2}$$

Or, it should be selected as being representative of the collection of values available in the literature. The consensus values for anisotropy function and radial dose function for most source models are mostly selected from Monte Carlo results that spanned the required range of radial distances and angles, and had sufficiently fine spacing to make the interpolation between points accurate.

The simulations were performed up to $1\times 10^{9}$ histories in water with statistical uncertainties of 0.05% to 0.1% at 1 and 5 cm on the transverse plane, and 2.5% and 3.5% at 1 and 5 cm along the long axis. In air with $7\times 10^{7}$ histories, statistical uncertainty was 1%.

## III. RESULTS & DISCUSSION

The MCNP simulation method in this work was benchmarked with the Theragenics model 200 palladium source. The comparison of calculated value of Λ for the model 200 in this study, $0.685\pm 0.021\;\text{cG}\cdot h^{-1}\cdot U^{-1}$, with the previously published data for the seed^(^ ^4^ ^)^ $0.686\;\text{cGy}\cdot h^{-1}\cdot U^{-1}$ (~0.1% difference), demonstrates the accuracy of our simulation method (Table 1). Also this table presents the consensus dose rate constants obtained for this source and other commercial sources. The measured values of type A uncertainty, $\%u_{A}(k=1)$ are approximately 2.9% and Monte Carlo calculation values of $\%u_{A}$ are approximately 2.1%. As shown in Sec. IV C of the TG‐43U1, the combined standard uncertainty is calculated by taking the square root of the sum of the squares of type A and type B uncertainties. Type B uncertainties are largely depended on source activity distribution inside the titanium capsule, on behavior and specification of the instruments which are evaluated by the scientific experience, and on historical data from the other similar measurements.

**Table 1:** Comparison of dose rate constant, Λ, of the IR06‐P103d seed with the measured and calculated values of model MED3633, Theragenics200, and Best double‐wall sources.

*Source Type*	*Method*	*Medium*	$\Lambda (cGy\cdot h^{-1}\cdot U^{-1})$	*Reference*
IR06‐P103d	Monte Carlo simulation (MCNP5)	Liquid water	0.692±0.021	(Present work)
	Monte Carlo simulation (MCNP5)	Perspex	0.691±0.021	(Present work)
	TLD dosimetry	Perspex	0.689±0.058	(Present work)
	Consensus value	Perspex	0.690	(Present work)
Theragenics 200	Monte Carlo simulation (MCNP5)	Liquid water	0.685±0.021	(Present work)
	Monte Carlo simulation	Liquid water	0.686±0.03	19
	TLD dosimetry	Solid water	0.650±0.08	21
MED3633	Monte Carlo simulation	Liquid water	0.677±0.02	22
	TLD dosimetry	Liquid water	0.680±0.05	20
Best Double‐wall	TLD dosimetry	Solid water	0.69±0.08	23
	Monte Carlo simulation	Liquid water	0.67±0.02	23

**Figure 3:**
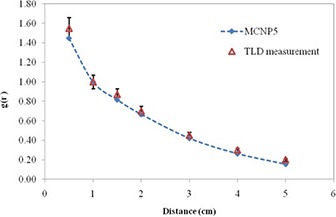
Comparison of the measured and the Monte Carlo‐calculated radial dose function of the IR06‐P103d source in Perspex phantom. The error bars indicate ±5% uncertainty in the measurement.

The measured and calculated radial dose functions, g(r), for the IR06‐P103d source in Perspex are shown graphically in Fig. 3. These, together with the values of the radial dose function for distances in the range 0.5–5 cm calculated in water, are tabulated in Table 2.

**Table 2:** Measured and calculated radial dose function of the IR06‐P103d source in Perspex phantom and also calculated in water.

	$g_{L}(r)$ *(Perspex)*		$g_{L}(r)$ *(Water)*
*r (cm)*	*MCNP5*	*TLD measurement*	*MCNP5*
0.5	1.448	1.49	1.42
1	1.000	1.000	1.000
1.5	0.817	0.841	0.822
2	0.668	0.689	0.668
3	0.422	0.431	0.426
4	0.264	0.276	0.264
5	0.156	0.168	0.161

**Figure 4:**
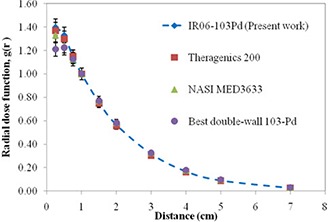
Comparison of the consensus radial dose function value of IR06‐P103d seeds versus three other available sources.^(^ ^4^ ^,^ ^23^ ^)^

Nominal uncertainty (one standard deviation) in the $g_{L}(r)$ data for the IR06‐P103d source is approximately 5% (ranging from 2% to 7%), shown as error bars in Fig. 3. The differences between the measured and calculated values of $g_{L}(r)$ are large depending on the distance. Measurements may be in error due to inadequate energy response or due to the finite dimensions of the TLDs used in this study. The total uncertainty due to the TLD measurement (type A and type B) as the energy response correction, TLD calibration, Perspex to liquid water phantom conversion, and repetitive TLD measurement is estimated to be about 7%. The radial dose function, $g_{L}(r)$, for the IR06‐P103d source and other commercial sources is included in Fig. 4.

A fifth‐order polynomial fit into the MCNP radial dose function in water for a range of r values from 0.5 to 5 cm yielded the following relationship: $g_{L}(r)=a_{0}+a_{1}r+a_{2}r^{2}+a_{3}r^{3}+a_{4}r^{4}+a_{5}r^{5}$, where $a_{0}=2.43E+00$, $a_{1}=-2.81E+00$, $a_{2}=2.02E+00$, $a_{3}=-7.66E-01$, $a_{4}=1.39E-01$, and $a_{5}=-9.48E-03$, define $R^{2}=0.999$.

**Figure 5:**
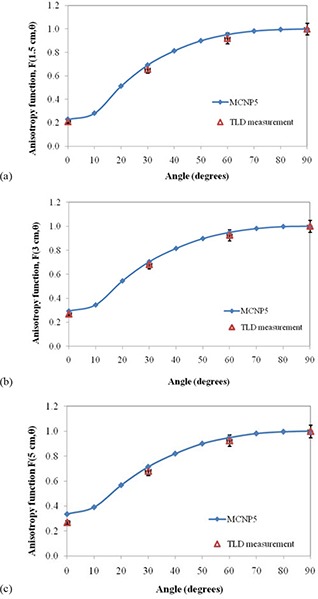
Comparison of the calculated anisotropy function of the IR06‐P103d seed versus measured values in Perspex phantom at radial distances of: (a) 1.5 cm, (b) 3 cm, (c) 5 cm.

The anisotropy function, F(r, θ), of the IR06‐P103d source was measured at radial distances of r=1.5,2,3, and 5 cm relative to the seed center, and polar angles θ ranging from 0° to 330° in 30° increments with respect to the seed long axis. For comparison, the Monte Carlo simulations were performed in Perspex at the same distances and angles as experimental measurements. The calculated and measured values for anisotropy function are presented in Table 3 and compared graphically at distances of r=1.5,3 and 5 cm in Fig. 5. A slightly difference between Monte Carlo calculated anisotropy function and measured values at shorter distances and small angles has been attributed to the large gradient of radiation in this angular range due to oblique filtration of the photons through the capsule and marker and also the thickness of the end caps.

**Table 3:** Measured and calculated anisotropy function, F(r,θ), for IR06‐P103d seed in Perspex.

							*F(r,* θ*)*						
	*MCNP5*										*TLD Measurement*		
*r (cm)*	*0*°	*10*°	*20*°	*30*°	*40*°	*50*°	*60*°	*70*°	*80*°	*90*°	*0*° *30*°	*60*°
0.25	0.053	0.074	0.616	0.854	0.924	0.959	0.979	0.992	0.998	1.000		
0.5	0.132	0.165	0.472	0.701	0.845	0.927	0.964	0.985	0.996	1.000		
1	0.190	0.242	0.491	0.686	0.816	0.903	0.956	0.982	0.996	1.000		
1.5	0.229	0.281	0.512	0.692	0.813	0.899	0.952	0.982	0.995	1.000	0.209 0.656	0.920
2	0.265	0.309	0.526	0.696	0.814	0.896	0.950	0.982	0.996	1.000	0.253 0.675	0.921
3	0.294	0.345	0.545	0.705	0.815	0.897	0.949	0.981	0.997	1.000	0.270 0.681	0.925
4	0.319	0.369	0.558	0.709	0.818	0.895	0.948	0.979	0.995	1.000		
5	0.337	0.392	0.568	0.715	0.820	0.900	0.948	0.981	0.995	1.000	0.301 0.682	0.905

**Figure 6:**
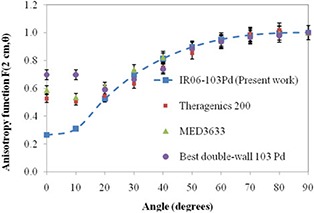
Comparison of the consensus anisotropy function values of the IR06‐P103d seed versus other available sources at 2 cm.^(^ ^4^ ^,^ ^23^ ^)^

Figure 6 shows the comparison of the anisotropy function of the IR06‐P103d source with several other commercial sources. The higher anisotropy functions for angles less than 20° for the MED3633 (Brachytherapy Services Inc., Springfield, VA; formerly, NASI), Theragenics 200, and Best double‐wall (Best Industries Inc., Springfield, VA), P103d sources were attributed to their thinner end caps and also to the distribution of radioactive material within these sources.

## IV. CONCLUSIONS

Dosimetric parameters including dose rate constant, Λ, radial dose function, g(r), and anisotropy function, F(r,θ) of the IR06‐P103d model brachytherapy seed have been measured in Perspex phantom using the corrected responses of TLD‐GR200A chips, according to the AAPM TG‐43U1 recommendations. The results were compared with calculated values for the same seed and also with three other commercial sources. The information is presented in tabulated and graphical format. The consensus value of dose rate constant for the IR06‐P103d source estimated to be $0.690\;\text{cGy}\cdot U^{-1}\cdot h^{-1}$ which is comparable with the published data of 0.686±0.003 for Theragenics 200 and 0.677±0.003 for MED3633.^(^ ^19^ ^,^ ^20^ ^)^ The experimentally defined radial dose functions and anisotropy functions also agree within the experimental uncertainties with corresponding MC data, confirming the latter for use in clinical practice. The radial dose function values measured in this study differ from the calculated values up to 7% at 5 cm away from the source. Measurement may be in error due to the inaccuracy in positioning and the unsuitable size of TLDs. Recommended TLD detector chips for dosimetry at distances <2 cm are $1\times 1\times 1\;{\text{mm}}^{3}$ and at distances ≥2 cm are $3\times 3\times 0.9\;{\text{mm}}^{3}$.^(^ ^7^ ^)^

The comparison of the calculated and measured parameters showed good agreement. Also the dosimetric parameter values of IR06‐P103d are in acceptable agreement with three other commercial source models, Theragenics 200, MED3633, and Best double‐wall. These results confirm this brachytherapy source has an acceptable dose distribution.

## ACKNOWLEDGMENTS

This research was supported by WCU (World Class University) program through the National Research Foundation of Korea funded by the Ministry of Education, Science and Technology (R31‐2008‐10029).
